# Improving Asthma Management: Patient–Pharmacist Partnership Program in Enhancing Therapy Adherence

**DOI:** 10.3390/pharmacy10010034

**Published:** 2022-02-17

**Authors:** Tatiana Makhinova, Jamie C. Barner, Carolyn M. Brown, Kristin M. Richards, Karen L. Rascati, Arpita Nag

**Affiliations:** 1 Faculty of Pharmacy and Pharmaceutical Sciences, University of Alberta, Edmonton, AB T6G 2E1, Canada; 2 College of Pharmacy, The University of Texas at Austin, Austin, TX 78712, USA; jbarner@austin.utexas.edu (J.C.B.); cmbrown@austin.utexas.edu (C.M.B.); krichards@austin.utexas.edu (K.M.R.); krascati@mail.utexas.edu (K.L.R.); 3 Sanofi Genzyme, Cambridge, MA 02142, USA; arpita.nag@sanofi.com

**Keywords:** asthma, community pharmacist, medication adherence, counseling tool, patient-centered care

## Abstract

Community pharmacist interventions can assist in improving adherence in patients with asthma. The objective of the study was to assess the feasibility of patient-centered counseling using the developed asthma-specific tools to identify barriers to adherence and identify their preliminary effect on adherence barrier score and asthma control. Adult patients with persistent asthma were invited to participate in a 3-month pre–post intervention study involving community pharmacist-provided patient-centered counseling. Bivariate analyses were conducted to determine whether there were changes in outcomes from the pre to post period. Of 36 recruited patients, 17 completed both pre and post surveys. At baseline, patients had a mean ACT score of 15.1 ± 3.5, with 94% having uncontrolled asthma, and an average of 4.2 ± 2.5 reported barriers. The following barriers were most common: not having an Asthma Action Plan (52.9%), use of inhaler more or less often than prescribed (47.1%) and forgetfulness (41.2%). The ACT score increased by 2.7 ± 5.4, which was not statistically significant; however, it might be clinically significant. Two barrier scores improved as a result of the intervention. Preliminary evidence on the feasibility of identifying and addressing patient-specific barriers to adherence delivered by pharmacists showed that it has the potential to resolve barriers and improve asthma outcomes.

## 1. Introduction

The lack of agreement between medical advice and patient behavior contributes significantly to uncontrolled asthma, which is associated with emergency department (ED) visits, hospitalizations, decreased quality of life and loss of productivity [1,2]. The prevalence of asthma attacks has not decreased over time, and 45.6% of all asthma patients had at least one asthma attack annually in the US [3]. Overall, 24% of exacerbations and 60% of asthma-related hospitalizations can be attributed to poor adherence [4]. However, medication adherence to controller therapy is consistently low, ranging between 30% and 70% overall and from 30% to 40% in “real-world” practice settings [5,6,7,8,9,10]. Therefore, addressing adherence to controller medications is critical in asthma management. 

Reasons for nonadherence vary greatly, and in addition to general barriers to adherence (e.g., forgetfulness), there are asthma-specific barriers such as lack of knowledge about appropriate inhaler use and misconceptions about asthma chronicity [8,11,12]. Therefore, interventions to improve adherence to asthma medications need to be tailored to individual patient-level factors, as they set a personalized goal in the patient’s mind [13]. Among healthcare professionals, community pharmacists are highly accessible and can communicate with and educate patients about effectively managing their asthma. Interventions conducted in pharmacy settings have been successful and have positively impacted controller therapy adherence; however, previously utilized interventions have tended to be complex, labor-intensive and time consuming, which can limit implementation in community pharmacies as well as other practice settings [4,14,15,16,17]. The literature shows that most of the patient-centered asthma programs with the goal of improving adherence have been conducted in Europe or Australia, whereas few studies have been conducted in the US. Because pharmacists have time and workflow barriers to the provision of patient-focused education and counseling, there is a need to equip pharmacists with efficient tools that will enhance patient–provider encounters [18]. Little is known about the effectiveness of individualized counseling based on identified barriers to adherence in asthma. In previous studies, the most common discussion points addressed during counseling sessions were the Asthma Action Plan, medication assessment, inhaler technique and symptom frequency, whereas there is little evidence regarding the inclusion of other adherence barriers, such as those that are patient- or provider-related [14,16,19,20]. 

Patient education can be delivered using a variety of strategies, but to be more efficient, patients’ needs should be identified first. When patients’ concerns and barriers are better understood, the intervention can be tailored (as opposed to general counseling), which has been shown to positively impact effectiveness [21]. This highlights the importance of patient-tailored education, and as evidence of this, one study showed that patients were dissatisfied with education that was not individualized [22].

Therefore, there is a need for more evidence on the effectiveness and feasibility of patient-centered asthma-specific counseling provided by pharmacists. Establishing an effective pharmacist-led program that will enhance asthma therapy adherence by identifying patient barriers and resolving them using patient-focused strategies will be an important step in addressing this need. The objective of this study was to assess the feasibility of the pharmacist-led program in improving adherence. Specifically, we aimed to determine if the identification of adherence barriers and patient-centered counseling using developed asthma-specific tools lead to an improved adherence barrier score and asthma control.

## 2. Materials and Methods

**Figure 1:**
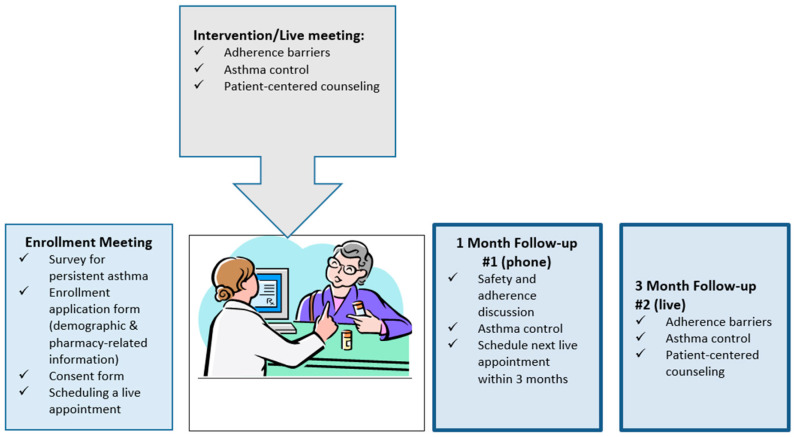
Intervention process.

This was a pilot feasibility study with a prospective, noncontrolled, pre–post evaluation of the intervention using patient surveys. Adult patients (≥18 years) with persistent asthma who filled their asthma controller medications in community pharmacies in urban central Texas were invited to participate in the study (Figure 1). 

To identify patients with persistent asthma, pharmacists asked patients two screening questions: “Has your doctor suggested that you use your inhaler regularly?” and “Do you have asthma symptoms most of the time or is it just a seasonal condition?” Patients who responded “Yes” to both were eligible for inclusion. Additionally, we were able to confirm persistent asthma based on the EPR-3 criteria developed by Schatz et al. [23]. The survey included 4 “Yes/No” items on impairment asking whether in the past 30 days, the patient had (1) “experienced asthma symptoms at least 3 times per week”, (2) “used (your) rescue inhaler for symptoms at least 3 times per week”, (3) “been awakened by asthma symptoms at least 3 times” and (4) experienced asthma that “interfered with the normal activity”. It also had a question assessing risk: (5) “In the past 12 months how many times have you been given oral corticosteroids (such as prednisone, methylprednisolone, medrol) for a flare up of your asthma?” Finally, it asked whether a patient had been taking asthma controller medication every day for the last 30 days. If a patient replied “yes” to one or more yes/no questions or had ≥ 2 oral corticosteroids (OCS) dispensed for an asthma flare indication in the past 12 months, persistence of asthma was indicated [23]. The first four yes/no questions from the persistency questionnaire asked about the same measures as the Asthma Control Test (ACT) questionnaire with a difference in the response scale. For example, EPR-3 persistence question: “Over the past 30 days, have you had asthma symptoms at least 3 times per week?” (Response: Yes, No); ACT question: “During the past 4 weeks, how often have you had shortness of breath?” (Response: More than once a day, Once a day, 3 to 6 times a week, Once or twice a week, Not at all). As can be seen, these two questions are compatible, and the answer to the EPR-3 persistence questionnaire can be derived from the ACT questionnaire (Appendix A: Survey #1, questions 2, 4, 6 and 7). The fifth EPR-3 persistence question regarding the use of oral corticosteroids was added to Survey #1 (Appendix A, question 11).

In addition to adult age and persistent asthma, other inclusion criteria were: (a) having a prescription for an asthma control inhaler, (b) willing to provide consent and personal contact information, (c) willing to be contacted via phone by the pharmacist for a 1-month post-initial follow-up and (d) willing to be contacted for a 3-month in-person follow-up with the pharmacist. If all criteria were met, then a consent form was signed, followed by the intervention. The participants who completed the study received two USD 25 gift certificates (baseline and 3-month follow-up). 

Patients were recruited by study pharmacists (N = 7) between July 2015 and February 2016. Four pharmacists were from four grocery store pharmacies, and three were from two community health center pharmacies. To expedite recruitment of patients, pharmacists queried their database to target patients who were due to pick up asthma controller inhaler prescriptions. Additionally, pharmacists advertised through signage in the pharmacy and fliers in bags of patients who used controller therapies. 

The survey instrument (Appendix A) included the following components: (a) demographic characteristics and comorbidities (5 items), (b) Asthma Control Test (ACT) (5 items) and (c) asthma-modified ASK-12 adherence tool (14 items). The outcome measures (dependent variables) were asthma control and adherence barrier score. The independent variable was study period (pre vs. post). Covariates included demographic information (gender, age, race/ethnicity and education) and chronic conditions. 

Asthma control was assessed with the Asthma Control Test (ACT), a reliable and valid test that has five questions with a response scale ranging from 1 (poor control) to 5 (good control) and a total score range of 5–25. Both continuous and dichotomous ACT scores (>19 (controlled) and ≤19 (uncontrolled)) were used [24,25]. Adherence barriers were assessed using a modified version of Adherence Starts with Knowledge (ASK)-12, an established generic instrument that identifies barriers to adherence [26]. ASK-12 is a reliable and valid (Cronbach’s alpha = 0.75) instrument designed to identify current medication adherence and patient-specific barriers. It is a 12-item patient survey that focuses on three subdomains that represent the most salient barriers to adherence: (1) inconvenience/forgetfulness, (2) treatment beliefs and (3) behavior. Modifications to the survey included changing “medication” to “inhaler” and adding two additional items pertaining to: having an Asthma Action Plan (AAP) and believing that controllers are for symptom-based use vs. continual use. The following scoring was used with the modified ASK instrument: items 1–9 with a 5-point Likert scale (1 = strongly disagree; 5 = strongly agree), items 10–14 with a 5-point scale (1 = in the last week; 5 = never) and items 4–8 with reverse scoring. The survey items were scored, and responses to each item signal where the patient experienced the most problems with adherence were identified. Subscale mean scores were created by taking the total subscale score and dividing it by the total number of items in the subscale. Likert scale responses of “4” or “5” for items 4–8 or “1” or “2” for items 1–3 and 9–14 were assigned a “1” for barrier presence; otherwise, they were assigned a “0”. The total was summed to derive the total number of barriers with the range 0–14. The barrier score range is 14–70, with a higher score indicative of more barriers. 

Two tools were developed during an earlier phase of this project: an “Asthma Conversation Starter” booklet for pharmacists and a “Breathe Easier” pamphlet for patients [27]. Based on the Drug Adherence Work-up (DRAW) tool, the “Asthma Conversation Starter” provides pharmacists with guidance on how to discuss and resolve adherence-related issues [28]. The DRAW tool was instrumental in helping pharmacists address patients’ barriers related to nonadherence because for each barrier, the tool provides practical approaches and suggestions to address the barrier [28]. Additionally, to ensure that the tools are patient-centered, once the barriers are identified, pharmacists further probe patients. For example, if patients have problems refilling their medications on time, the “Asthma Conversation Starter” tool prompts pharmacists to inquire further regarding potential issues such as cost, access to primary care, transportation or need for reminders. 

The aim of the current study was to pilot the developed tools and assess the preliminary impact of the intervention on the adherence barrier score and asthma control with patients who have persistent asthma and who use community pharmacies. Pharmacists were trained by the principal investigator on obtaining informed consent, administering the surveys and utilizing the developed counseling tools. Practice sessions and performance evaluation prior to implementation were not part of the training process. Each pharmacist was asked to recruit at least 5 adult patients meeting the inclusion criteria. The first in-person appointment (30–40 min) included time for patients to review the informed consent (15–20 min), complete the survey (identifying barriers to adherence, asthma control, demographics and comorbid conditions) and receive pharmacist counseling (15–20 min). Pharmacists identified reasons for nonadherence based on the survey and further probing and provided patient-specific education to resolve adherence issues (using the counseling tools “Asthma Conversation Starter” and “Breath Easier”). Action plans and recommendations were developed for each patient. At the 1-month telephone follow-up, the pharmacist inquired about the resolution of the patient’s adherence barriers and asked if there were questions or concerns. During the 3-month in-person follow-up (15–20 min) as a part of the dispensation process of refill medication(s), a pharmacist assessed barriers to adherence and asthma control based on information from the patients’ follow-up survey. Pharmacists also reinforced suggested recommendations regarding medication-taking behaviors, lifestyle, adherence and asthma control. 

Data from patients were collected via surveys during the 2 sessions with the pharmacists: (1) initial in-person meeting and (2) 3-month in-person post-initial follow-up. Once all surveys were completed, the data were consolidated into an Excel spreadsheet using a codebook. Descriptive statistics included means (SD), medians (IQR) and frequencies. Paired bivariate analyses (Wilcoxon signed-rank tests and paired t-tests) were run to determine whether there were changes in outcomes from the pre to post period. Data were analyzed using SAS, version 9.4 (Cary, NC, USA). Institutional Review Board approval was granted prior to study initiation. 

## 3. Results

Of 36 patients who were recruited to participate in the study, 17 patients completed both pre and post surveys and were eligible for analysis. Table 1 displays baseline patient characteristics for both those who completed the study and those who dropped out of the study. Based on the feedback that we received from the participating pharmacists, those who dropped out mainly refused to participate in the 3-month follow-up. Interestingly, those who dropped out of the study had significantly better asthma control. Those who completed the study had the following characteristics: mean age was 41.7 ± 16.6 years, with 58.8% females, 41.2% Caucasian and 17.6% Hispanic/Latino. The majority (64.7%) of participants had some college/college degree or higher, and 35.3% reported asthma as their only chronic condition. The mean asthma control score was low (15.1 ± 3.5), with 94.1% having uncontrolled asthma (ACT ≤ 19). The mean barrier to adherence score was 31.2 ± 7.2. 

All participants (N = 17) had at least one barrier to adherence (i.e., responded “4” or “5” on the Likert scale for items 4–8 and “1” or “2” for items 1–3 and 9–14), and the mean number of barriers in the pre-intervention period was 4.2 ± 2.5 (Table 1). The following barriers were most commonly reported: item 6: not having an AAP and not reaching goals (52.9%); item 10: use of inhaler more or less often than prescribed (47.1%); item 1: forgetfulness (41.2%); item 9: use of controller inhaler only when symptoms were present (35.3%); and item 14: not having an inhaler when it was time to use it (35.3%) (Table 2). Out of the three subscales, inconvenience/forgetfulness had the highest median subscale score (2.3 (1.0)), closely followed by treatment beliefs (2.3 (0.5)). Paired t-tests revealed that the following individual barriers decreased significantly from pre- to post-intervention: possession of AAP (4.0 (1.0) to 2.0 (1.0)); frequency change of barrier being present (52.9% vs. 17.6%, respectively) and cooperative work with doctor/nurse (3.0 (1.0) to 2.0 (1.0)); frequency change of barrier being present (23.5% vs. 0%, respectively). The other individual barrier scores did not change. 

Table 3 shows that participants’ ACT scores increased from 16.0 (3.0) (pre) to 18.0 (8.0) (post), though not significantly (*p* = 0.060). Similarly, when examining the change in total barrier score from pre to post, barriers decreased from 30.0 (8.0) to 29.0 (10.0) (*p* = 0.053). When the scores for each of the three subscales were compared separately, the treatment beliefs barrier scale score decreased significantly (*p* = 0.008). 

## 4. Discussion

This study was designed to help patients with persistent asthma (self-reported) who utilize controller medications by improving adherence to treatment. Overall asthma management strategies in this study were implemented through a patient-centered approach of identifying and addressing barriers to adherence in community pharmacy settings. Utilizing the following developed intervention tools: patient survey to identify adherence barriers, pharmacist booklet “Asthma Conversation starter” to guide pharmacist-patient interactions and patient pamphlet “Breathe easier” to reinforce education provided, this study aimed to provide evidence on the feasibility and preliminary effect of the proposed intervention. Thus, barriers to asthma adherence and asthma control from the pre to post period were compared. 

The patient survey helped to reveal patient-specific barriers in a timely manner. We found that participants had at least one barrier, with a mean of 4.2 ± 2.5. At baseline, the reported barriers included items from all three domains, which signal a variety of possible reasons for nonadherence; hence, the recognition of specific barriers for patients may be an important step for implementing targeted counseling. This was also consistent with the results of a cross-sectional study using the same patient survey [29]. 

The most frequently reported adherence barrier item pertains to the treatment beliefs subscale and refers to the possession of an AAP and knowing the goals of asthma management. More than half of participants reported this barrier, which was also found in other studies [14,30]. Surprisingly, several studies examining physicians’ perceptions regarding the need for an AAP revealed that they felt that the AAP was not required [31,32,33]. Perhaps a lack of physician endorsement leads to patients not receiving AAPs from providers; however, research indicates that setting goals for patients is an important step for asthma improvement [14]. Thus, encouraging physicians to provide and patients to obtain an AAP and to set goals may lead to improvements in adherence. 

Four additional prevalent barriers to adherence were (1) forgetfulness, (2) symptom-based use of controller inhalers, (3) not using inhaler as prescribed and (4) not having the inhaler when needed. Although these were identified at baseline, there were no significant changes in the follow-up analysis. Forgetfulness is a common reason for nonadherence among patients with asthma; however, in this study, participants were encouraged to be proactive by taking actions on their own to overcome this barrier through suggested strategies (e.g., getting reminder apps or signing up for text message reminders). Potentially, more advanced technologies, such as automated phone calls and audiovisual reminder devices, should be implemented, as they have been shown to positively impact adherence [34]. The misconception that controller inhalers should only be used when symptoms are present is well documented [12,35]. It is also evident that with the provision of education by pharmacists, patients can better understand asthma, as well as how to use their medications appropriately [21]. Patient education should be addressed with a focus on understanding the difference between rescue and controller inhalers [8,35,36]. The positive patient response regarding “not using inhaler as prescribed” reiterates the presence of the barrier to adherence, and pharmacists should probe further to determine specific causes if not addressed in the survey. When a patient’s nonadherence is due to “not having an inhaler when needed”, pharmacists should work with the patient to collaboratively develop strategies for having access to an inhaler.

The study findings demonstrate that two barriers were resolved over a course of 3 months after the intervention. Regarding the first barrier, two-thirds of participants without AAPs and awareness about their asthma management goals at the baseline (N = 9) were found to report having an AAP and being aware of the goals at the 3-month follow-up (N = 6). The pharmacists were proactive in providing patients with AAPs, or they referred patients to their primary care providers to obtain AAPs. In previously conducted studies, the provision of written information and education was shown to improve controller inhaler adherence [37,38]. Regarding the second barrier, all patients (N = 4) who disagreed that they “work cooperatively with their doctor/nurse to make decisions” at the baseline indicated that they did so at the 3-month follow-up. As nonadherent patients demonstrate a lack of knowledge and misperceptions regarding medications [39], communication between healthcare providers and patients, together with a cooperative approach, may result in improvements in knowledge and skills, which may lead to positive outcomes. Based on the improvement in responses about this barrier from baseline to follow-up, we can assume that our approach seemed to be successful in promoting team-based care. 

Although we anticipated observing significant improvements in asthma control, this was not the case in our study. The score of asthma control increased by 2.7 points, which was not statistically significant but was clinically meaningful, as the minimally important difference in ACT score is between 2 and 3 [23]. The clinically meaningful change of ~3 points may lend support to the effectiveness of the patient-centered barrier resolution approach to asthma control. In two studies evaluating the effectiveness of similar programs, asthma control improved significantly at the 12-month follow-up [17,37]. Perhaps with a longer follow-up and larger sample size, our study results would be similar. Additionally, we identified that participants who dropped out of the study had significantly better asthma control, which might explain their disinterest in following up with the pharmacists. It is important to further investigate who should be targeted for the intervention in order to benefit more from the intervention and better cooperate with pharmacists. Another possible explanation of the high dropout rate may be a lack of relationship between participants and their pharmacists; specifically, patients may not see them as continuous healthcare providers. 

The study findings did not show significant improvement in total adherence barrier scores between pre and post periods. From the three separate subscales, “treatment beliefs” was the only one with significant improvement from the pre to post period. This highlights the need to focus on beliefs in asthma management, as barriers related to beliefs are associated with problematic disease control [40]. The most commonly reported patient health beliefs (necessity beliefs, concern beliefs and knowledge of medications) and social support that impact adherence are reflected in the ASK-12, which makes it a valuable tool to use [8,11]. 

This study was subject to several limitations. First, practice sessions and performance evaluation prior to implementation were not part of the pharmacists’ training. However, the tools, including surveys, were developed in collaboration with practicing pharmacists [27]. Second, implementation fidelity was not formally measured; however, the research team contacted pharmacists either via phone or in person every 1–2 weeks to make sure that the implementation was going according to the plan. No significant issues were raised by the pharmacists during these regular meetings. Third, this study did not have a control group, which might affect the reported association between the intervention and the outcomes. Fourth, this pilot study had a small sample size (approximately 50% of the original sample was present in the follow-up) with a short follow-up period, which means that with larger sample sizes (which will increase power) for follow-up, the results may differ. Additionally, the inability to control for confounders in the statistical analysis could also have affected the results. Fifth, self-reported barriers to adherence, persistence of asthma and asthma control may be a source of recall bias. This bias could be resolved by coupling self-reported measures with objective measures such as pharmacy claims to calculate adherence using the proportion of days covered or medical claims to verify asthma control. In this study, we did not have access to the pharmacy claims data. In addition, questions revealing barriers to adherence asked about the patient’s experiences, beliefs and perceptions rather than asking them to recall specific events or frequencies. Sixth, another study limitation is selection bias, as this study examined barriers to adherence among a convenience sample of patients who were picking up their prescription for asthma medication. Seventh, participants were from urban central Texas community pharmacies, and only a small proportion of the sample was not college educated and reported cost as a barrier to adherence. Consequently, the results from this patient sample may not be generalizable to a broader population of asthma patients across geographic and socioeconomic characteristics or social groups, as well as the underserved. Finally, in order to capture changes in asthma control, a longer prospective observation and data collection period might be needed, as 3 months may not be adequate to assess the impact of the intervention. 

## 5. Conclusions

The results from this pilot study show that a tailored approach of identifying and addressing patient-specific barriers to adherence delivered by pharmacists can be effective in resolving adherence barriers. This approach also has the potential to improve asthma control, as a potentially clinically meaningful ACT score change of almost 3 points occurred in a 3-month period. The findings support the need for a targeted approach in asthma care, as reasons for nonadherence vary among patients. Individually targeting patient needs during counseling may yield improved outcomes. Future research is needed to test the effectiveness of utilizing these tools in pharmacy practice on a larger scale.

## Figures and Tables

**Table 1:** Baseline characteristics.

Variable	Respondents (N = 17)		Non-Respondents (N = 19)		*p*-Value
Age (years), mean (SD)	41.7	16.6	38.9	13.3	0.58
	**N**	**%**	**N**	**%**	
Female	10	58.8	14	73.7	0.34
Race/ethnicity					N/A ^1^
Caucasian	7	41.2	12	63.2	
African American	2	11.8	2	10.5	
Hispanic/Latino	3	17.6	5	26.3	
Other	5	29.4	-		
Level of education					N/A ^1^
Primary	-		1	5.3	
Some high school	3	17.6	1	5.3	
High school	3	17.6	2	10.5	
Some college	5	29.4	3	15.8	
College	4	23.5	8	42.1	
Postgraduate	2	11.8	4	21.0	
Chronic condition(s)					N/A ^1^
None ^2^	6	35.3	7	36.8	
Hypertension	4	23.5	6	31.6	
Diabetes	1	5.9	2	10.5	
Dyslipidemia	1	5.9	4	21.0	
Other ^3^	8	47.1	11	57.9	
ACT, mean (SD)	15.1	3.5	19.4	3.7	0.001
Control level					0.001
≤19 (uncontrolled)	16	94.1	8	42.1	
>19 (controlled)	1	5.9	11	57.9	
Barrier to adherence score, mean (SD) ^4^	31.2	7.2	28.7	7.9	0.33
Number of barriers, mean (SD)	4.2	2.5	3.7	2.3	0.5

ACT = Asthma Control Test. ^1^ Cell sizes too small for analysis. ^2^ Other than asthma. ^3^ Depression, chronic pain, gastrointestinal disorder, thyroid disease, heart disease, arthritis, obesity, allergies, multiple sclerosis or osteoporosis. ^4^ Barrier score range: 14–70; higher score = more barriers.

**Table 2:** Comparison of differences in barriers to adherence from pre to post period (N = 17).

	Pre Period		Post Period		*p*-Value ^3^
Individual Barriers	Median ^1^ (IQR)	Barrier Present ^2^ N (%)	Median ^1^ (IQR)	Barrier Present^2^ N (%)	
I just forget to use my inhaler some of the time	2.0 (3.0)	7 (41.2%)	2.0 (2.0)	6 (35.3%)	0.8174
2. I run out of my inhaler because I don’t get refills on time	2.0 (1.0)	4 (23.5%)	2.0 (2.0)	4 (23.5%)	0.9863
3. Using my inhaler more than once a day is inconvenient	2.0 (2.0)	5 (29.4%)	2.0 (2.0)	5 (29.4%)	0.9844
4. I feel confident that my inhaler will help me	2.0 (1.0)	1 (5.9%)	1.0 (1.0)	1 (5.9%)	0.6250
5. I know how to use my inhaler correctly	1.0 (1.0)	1 (5.9%)	1.0 (1.0)	1 (5.9%)	1.0
6. I have an Asthma Action Plan and know if I am reaching my goals	4.0 (1.0)	9 (52.9%)	2.0 (1.0)	3 (17.6%)	0.0034
7. I have someone I can call with questions about my inhaler	2.0 (1.0)	3 (17.6%)	2.0 (0)	1 (5.9%)	0.0972
8. My doctor/nurse and I work together to make decisions	3.0 (1.0)	4 (23.5%)	2.0 (1.0)	0 (0%)	0.0010
9. I only use my inhaler when I am having symptoms such as shortness of breath, coughing, wheezing, or chest tightness	2.0 (2.0)	6 (35.3%)	2.0 (3.0)	6 (35.3%)	0.9922
10. Used your inhaler more or less often than prescribed?	3.0 (3.0)	8 (47.1%)	1.0 (3.0)	7 (41.2%)	0.7500
11. Skipped or stopped using your inhaler because you didn’t think it was working?	1.0 (0)	1 (5.9%)	1.0 (0)	0 (0%)	0.7500
12. Skipped or stopped using your inhaler because it made you feel bad?	1.0 (0)	0 (0%)	1.0 (0)	0(0%)	1.0
13. Skipped, stopped, not refilled, or used less of inhaler because of the cost?	1.0 (0)	1 (5.9%)	1.0 (0)	0 (0%)	0.6250
14. Not had your inhaler with you when it was time to use it?	2.0 (3.0)	6 (35.3%)	1.0 (3.0)	6 (35.3%)	0.8232
**Total Barrier Scale Score ^4^**	30.0 (8.0)		29.0 (10.0)		0.0530

^1^ Individual barrier score range: 1–5; higher score = more barriers. ^2^ Likert scale response “4” or “5” for Items 4–8 or “1” or “2” for Items 1–3, 9–14. ^3^ Wilcoxon signed-rank test; bold indicates significance. ^4^ Barrier score range: 14–70; higher score = more barriers. 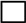 Inconvenience/forgetfulness subscale. 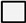 Treatment beliefs subscale. 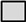 Behavior subscale.

**Table 3:** Comparison of asthma control and barrier score between participants in pre and post groups.

	Pre Median (IQR)	3-Month Post Median (IQR)	*p*-Value ^3^
ACT	16.0 (3.0)	18.0 (8.0)	0.060
**Barrier score**			
Overall ^1^	30.0 (8.0)	29.0 (10.0)	0.053
Subscales ^2^			
Behavior	1.8 (0.8)	1.6 (1.2)	0.370
Forgetfulness	2.3 (1.0)	2.3 (1.0)	0.772
Beliefs	2.3 (0.5)	2.2 (0.8)	**0.008**

ACT = Asthma Control Test: >19 (controlled); ≤19 (uncontrolled). ^1^ Barrier score range: 14–70; higher score = more barriers. ^2^ Subscale scores were adjusted for number of items in the subscale. ^3^ Bold indicates significance.

## Data Availability

The data presented in this study are available from the corresponding author upon request.

## Appendix A. Survey #1: Your Asthma Experiences

The following questions ask about your asthma symptoms. Please answer each question by marking ONE box. If you are unsure about how to answer, please give the best answer you can.

Do you know the difference between your ‘rescue’ and ‘controller’ inhaler?

☐ Yes

☐ No, STOP here and ask a pharmacist to explain it to you. Then proceed answering the remaining questions.

1. During the past 4 weeks, how often have you used your rescue inhaler or nebulizer medication (such as Ventolin^®^ or albuterol)?

☐ 3 or more times per day

☐ 1 or 2 times per day

☐ 2 or 3 times per week

☐ Once a week or less

☐ Not at all

2. I have difficulties using my inhaler(s).

☐ Strongly agree

☐ Agree

☐ Neutral

☐ Disagree

☐ Strongly disagree

3. During the past 4 weeks, how often did your asthma symptoms (wheezing, coughing, shortness of breath, chest tightness or pain) wake you up at night or earlier than usual in the morning?

☐ 4 or more nights a week

☐ 2 or 3 nights a week

☐ Once a week

☐ Once or twice a week

☐ Not at all

4. How would you rate your asthma control during the past 4 weeks?

☐ Not controlled at all

☐ Poorly controlled

☐ Somewhat controlled

☐ Well controlled

☐ Completely controlled

5. In the past 4 weeks, how much of the time did your asthma keep you from getting as much done at work, school or at home?

☐ All of the time

☐ Most of the time

☐ Some of the time

☐ A little of the time

☐ None of the time

6. During the past 4 weeks, how often have you had shortness of breath?

☐ More than once a day

☐ Once a day

☐ 3 to 6 times a week

☐ Once or twice a week

☐ Not at all

7. Have you EVER been hospitalized (at least overnight) due to your asthma?

☐ Yes

☐ No

8. In the past 12 months, how many times have you been hospitalized (at least overnight) for an asthma attack? (IF NONE WRITE “0”)

________________

9. In the past 12 months, how many times did you get treatment for an acute asthma attack at a doctor’s office, urgent care facility or emergency department (ER)? (IF NONE WRITE “0”)

__________________

10. In the past 12 months, how many times have you been given oral corticosteroids (such as prednisone, methylprednisolone, Medrol®) for a flare up of your asthma? (IF NONE WRITE “0”)

___________________

11. Do you have an Asthma Action Plan?

☐ Yes

☐ No

12. Mark one answer for every item below.

**Note: Inhaler below Refers to CONTROLLER Inhaler**	**Strongly** **Agree**	**Agree**	**Neutral**	**Disagree**	**Strongly Disagree**
I just forget to use my inhaler some of the time	☐	☐	☐	☐	☐
I run out of my inhaler because I don’t get refills on time	☐	☐	☐	☐	☐
Using my inhaler more than once a day is inconvenient	☐	☐	☐	☐	☐
I feel confident that my inhaler will help me	☐	☐	☐	☐	☐
I know how to use my inhaler correctly	☐	☐	☐	☐	☐
I have an Asthma Action Plan and know if I am reaching my goals	☐	☐	☐	☐	☐
I have someone I can call with questions about my inhaler	☐	☐	☐	☐	☐
My doctor/nurse and I work together to make decisions	☐	☐	☐	☐	☐
I only use my inhaler when I am having symptoms such as shortness of breath, coughing, wheezing, or chest tightness	☐	☐	☐	☐	☐

Have you…	**In the last week**	**In the last month**	**In the last 3 months**	**>3 months ago**	**Never**
Used your inhaler more or less often than prescribed?	☐	☐	☐	☐	☐
Skipped or stopped using your inhaler because you didn’t think it was working?	☐	☐	☐	☐	☐
Skipped or stopped using your inhaler because it made you feel bad?	☐	☐	☐	☐	☐
Skipped, stopped, not refilled, or used less of inhaler because of the cost?	☐	☐	☐	☐	☐
Not had your inhaler with you when it was time to use it?	☐	☐	☐	☐	☐

	**Every day**	**Once a week**	**Once a month**	**When asthma worsens**	**I don’t have one**
How often do you use your peak flow meter?	☐	☐	☐	☐	☐

13. Do you have any other chronic conditions? Please check all that apply.

☐ Hypertension	☐ Dyslipidemia	☐ Diabetes	☐ Depression
☐ Chronic Pain	☐ Gastrointestinal disorder	☐ Thyroid disorder	☐ Heart disease
☐ Arthritis	☐ Obesity	☐ Other, Please specify _______________________	

14. What is your gender?

☐ Male

☐ Female

15. What is your race/ethnicity?

☐ Caucasian

☐ African American

☐ Hispanic/Latino

☐ Other

16. What year were you born?

__________

17. What is the highest level of education you have completed?

☐ Primary

☐ Some high school

☐ High school

☐ Some college

☐ College

☐ Postgraduate (MS, MA, MBA, etc.)

Please return to the pharmacy staff. THANK YOU!
